# *Astragalus wuqiaensis* (Fabaceae, Papilionoideae, Astragaleae), a new species from Xinjiang, China

**DOI:** 10.3897/phytokeys.272.186990

**Published:** 2026-04-01

**Authors:** Si-Wei Zeng, Kai-Wen Jiang, Cheng-Wen Luo, Pan Li, Zhao-Ping Yang

**Affiliations:** 1 College of Life Sciences and technology, Tarim University, Aral, 843300, China Tarim University Aral China; 2 State Key Laboratory Incubation Base for Conservation and Utilization of Bio-Resource in Tarim Basin, Aral, 843300, China State Key Laboratory Incubation Base for Conservation and Utilization of Bio-Resource in Tarim Basin Aral China; 3 Key Laboratory of National Forestry and Grassland Administration on Plant Conservation and Utilization in Southern China, Guangzhou 510650, China Key Laboratory of National Forestry and Grassland Administration on Plant Conservation and Utilization in Southern China Guangzhou China; 4 South China National Botanical Garden, Guangzhou 510650, China South China National Botanical Garden Guangzhou China; 5 Ningbo Botanical Garden, Ningbo 315201, China Ningbo Botanical Garden Ningbo China; 6 Key Laboratory of Biodiversity and Environment on the Qinghai-Tibetan Plateau, Ministry of Education, School of Ecology and Environment, Xizang University, Lhasa, China Xizang University Lhasa China

**Keywords:** *
Astragalus
*, morphology, new taxon, phylogeny, Xinjiang Province

## Abstract

During an extensive botanical survey in Ulugqat Township, Wuqia County, Xinjiang, China—a region along the country’s western border that remains significantly understudied—*Astragalus wuqiaensis*, a new species in *Astragalus* sect. *Cenantrum* (Fabaceae) was discovered. This perennial herb is characterized by its free stipules, yellow flowers, lax racemes, a long-stipitate ovary, and membranous fruit valves, aligning with the diagnostic features of sect. *Cenantrum* and supporting its placement therein. It differs from other sect. *Cenantrum* members by its markedly smaller leaflets (2–8 mm × 2–5 mm). Phylogenetic analysis of nrITS sequences provides robust support for its sectional affiliation (BS = 94%, PP = 1). A detailed morphological description, diagnostic illustrations, and habitat information are provided to confirm its novelty.

## Introduction

The genus *Astragalus* L. (Fabaceae, subfam. Papilionoideae, tribe Astragaleae), established by Linnaeus in 1753, is the largest genus of angiosperms, encompassing over 3,100 accepted species classified into 255 sections (Maassoumi et al. 2020). With a nearly global distribution, excluding only Australia and New Zealand (POWO 2025), *Astragalus* species are predominantly herbaceous or semi-shrubby plants valued as forage resources (Lock and Schrire 2005). A few species hold significant economic importance: *A. gummifer* Labill. (gum tragacanth) provides gum for diverse industries; *A. membranaceus* Bunge and *A. mongholicus* Bunge are staples in traditional medicine; and other species (milk vetches) are used for human food (edible pods, although some are toxic), erosion control, fuelwood, and as indicators of selenium and uranium (Allen and Allen 1981; Lock and Schrire 2005).

The section *Cenantrum* Bunge [*Astragalus* subg. *Phaca* (L.) Bunge] comprises ca. 40 species and three subspecies, distributed primarily in northwestern China, Mongolia, and Russia (Xu and Podlech 2010; Maassoumi et al. 2020+). Ho and Fu (1993), in “Flora Reipublicae Popularis Sinicae”, recognized 24 species, one subspecies, and seven varieties of this section in China, whereas Xu and Podlech (2010) recognized 27 species in “Flora of China”. According to Ho and Fu (1993), species of this section are characterized by a perennial herbaceous habit with free stipules; flowers ranging from yellow to purplish-red arranged in lax racemes on peduncles 10–20 cm long; a stipitate ovary; and pods characterized by membranous valves and a unilocular interior.

Wuqia County, located in the westernmost part of China and bordering Kyrgyzstan, is a biogeographically important but floristically poorly known border region, where its remote location and difficult accessibility have led to considerable floristic knowledge gaps. The discovery potential of this region is highlighted by several recent findings: the new species *Ferula diversifolia* W.Jun Li & Lei Yang (Yang et al. 2025), the first record of *Corydalis pseudoadunca* Popov (Liu et al. 2025) for China, and our team’s first record of the genus *Colchicum* (Colchicaceae) in China (Zeng et al. in press). Furthermore, during a survey conducted in July 2024, we discovered and collected a morphologically distinctive *Astragalus* species that did not match any known taxa. Here, we integrate morphological and molecular phylogenetic evidence to confirm its identity as a new species within sect. *Cenantrum* and provide its formal description.

## Materials and methods

### Material collection, morphological observations, and identification

During a field survey in July 2024, this distinct taxon was encountered. Its habitat, growth form, and fresh vegetative and reproductive structures were photographed. Its specimens were collected (Voucher specimen: YZP001513), and young leaves were sampled and preserved in silica gel for subsequent DNA extraction and sequencing.

Morphological observations were conducted on fresh materials and preserved specimens with complete reproductive structures (flowers and fruits). Preliminary identification was performed by comparing the morphological characters with taxonomic treatments in “Flora of the USSR” (Borisova et al. 1946), “Flora of the Kirghiz SSR” (Nikitina 1957), “Flora Reipublicae Popularis Sinicae” (Ho and Fu 1993), and “Flora of China” (Xu and Podlech 2010), which indicated an affiliation within sect. *Cenantrum*. To verify this placement, we examined digitized specimens of this section accessed through several online herbarium platforms, including the Chinese Virtual Herbarium (CVH, https://www.cvh.ac.cn/), the Global Biodiversity Information Facility (GBIF, https://www.gbif.org/), the Vascular Plants Herbarium of the Komarov Botanical Institute (LE, https://en.herbariumle.ru/), and JSTOR Global Plants (https://plants.jstor.org/).

### Phylogenetic analyses

Whole-genome resequencing was carried out using the Illumina NovaSeq 6000 platform at Novogene (Tianjin, China). The sequencing generated 2 × 150 bp paired-end reads with a target depth of 20×, yielding a total of 9.65 GB of raw data. The complete nuclear ribosomal DNA (nrDNA) repeat unit was assembled using GetOrganelle v1.7.7.1 (Jin et al. 2020). The nrITS region was extracted using ITSx v1.1.3 (Bengtsson-Palme et al. 2013). Furthermore, leaf material of *A. zadaensis*—one of the two morphologically most similar species to *A. wuqiaensis*—was obtained from the Germplasm Bank of Wild Species (China). Its nrITS sequence was then generated following the aforementioned methods and included in the phylogenetic analysis. The other morphologically similar species, *A. hoffmeisteri*, could not be included in the molecular analysis due to the unavailability of material; accordingly, its morphological differences from *A. wuqiaensis* are presented in Table 2. For phylogenetic analysis, nrITS sequences from 25 *Astragalus* species—including all currently available species of sect. *Cenantrum* (14 species) in the National Center for Biotechnology Information (NCBI) (https://www.ncbi.nlm.nih.gov/)—were downloaded. Two species of the genus *Oxytropis* DC., another member of the tribe Astragaleae, *Oxytropis aciphylla* Ledeb. and *O. almaatensis* Bajtenov, were used as outgroups.

Sequence alignment was performed with MAFFT v7.525 (Katoh and Standley 2013), and the resulting alignment was trimmed using trimAl v1.5 (Capella-Gutiérrez et al. 2009) under the “automated1” setting to generate the final matrix. Maximum likelihood (ML) analysis was conducted in IQ-TREE v1.6.12 (Nguyen et al. 2015), with ModelFinder (Kalyaanamoorthy et al. 2017) selecting the best-fit model (K2P+G4) based on the Bayesian Information Criterion (BIC). Branch support was assessed using 1,000 bootstrap replicates. Bayesian inference (BI) was performed using MrBayes v3.2.7a (Ronquist et al. 2012) via PhyloSuite v2.0 (Zhang et al. 2020), employing the K2P+G4 model selected by the built-in ModelFinder. Two parallel runs of 10 million generations were conducted, sampling every 1,000 generations, with the first 25% of samples discarded as burn-in. Phylogenetic trees were visualized using iTOL v6 (Letunic and Bork 2024) (https://itol.embl.de).

## Results

### Molecular phylogeny

**Figure 1.**
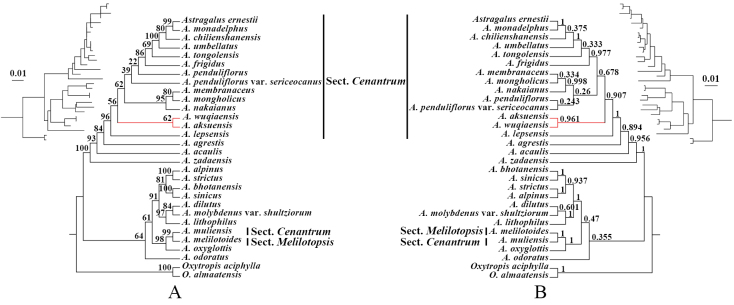
Phylogenetic trees based on nrITS sequences of representative *Astragalus* species. **A**. Maximum likelihood (ML) tree, with bootstrap support (BS) values shown on branches; **B**. Bayesian inference (BI) tree, with posterior probability (PP) values shown on branches.

Phylogenetic analysis of nrITS sequences placed *A. wuqiaensis* within a monophyletic clade comprising 13 species of sect. *Cenantrum* (BS = 96%, PP = 1.0). It formed a sister clade to *A. aksuensis* Bunge (BS = 62%, PP = 0.961), corroborating its morphological assignment to sect. *Cenantrum*. Furthermore, *A. zadaensis*, which is morphologically most similar to *A. wuqiaensis*, was not placed within the sect. *Cenantrum* clade, supporting the classification of *A. wuqiaensis* as a new species. Notably, *A. muliensis* Hand.-Mazz.—currently placed in sect. *Cenantrum*—clustered in a separate monophyletic clade with *A. melilotoides* Pall. of sect. *Meliotopsis* Gontsch (Fig. 1; Table 1).

**Table 1.** The nrITS sequence information used in the phylogenetic analysis (GenBank/NCBI), with taxon and accession numbers.

Taxon	Accession numbers
*Astragalus acaulis*	MF044257
*Astragalus agrestis*	PQ008000
*Astragalus alpinus*	HQ613380
*Astragalus aksuensis*	AF359753
*Astragalus bhotanensis*	MH808429
*Astragalus chilienshanensis*	MW432232
*Astragalus dilutus*	PQ492291
*Astragalus ernestii*	MH293126
*Astragalus frigidus*	AB231092
*Astragalus lithophilus*	MT923542
*Astragalus melilotoides*	HM142302
*Astragalus membranaceus*	AF121675
Astragalus molybdenus var. shultziorum	AF121693
*Astragalus monadelphus*	MH808432
*Astragalus mongholicus*	KT201426
*Astragalus muliensis*	MZ198530
*Astragalus nakaianus*	KC262198
*Astragalus odoratus*	LC529256
*Astragalus oxyglottis*	AB051932
*Astragalus penduliflorus*	PQ590582
Astragalus penduliflorus var. sericeocanus	PQ763906
*Astragalus sinicus*	MH808434
*Astragalus strictus*	KP338105
*Astragalus tongolensis*	MH293127
*Astragalus umbellatus*	AF121683
*Astragalus wuqiaensis*	PX931885
*Astragalus zadaensis*	PX901884
*Oxytropis almaatensis*	MG282028
*Oxytropis aciphylla*	GQ422806

### Taxonomic treatment

#### Astragalus wuqiaensis

Taxon classificationPlantae FabalesFabaceae

Z.P.Yang, S.W.Zeng & P.Li sp. nov.

679575FA-31AB-5D1A-AEB8-D531B743520F

urn:lsid:ipni.org:names:77378247-1

##### Diagnosis.

This species is characterized by erect stems forming dense clumps. The stems are densely covered with white stiff hairs at the base, becoming sparser upwards; the peduncles are nearly glabrous. Leaves are odd-pinnate, 4–8 cm long; leaflets 9–20 pairs, 2–8 mm × 2–5 mm, ovate to elliptic, glabrous adaxially, and with white short-appressed hairs abaxially. This set of characters readily distinguishes it from other species in sect. *Cenantrum*. Nevertheless, when examining herbarium specimens, it shows closer morphological affinity to *Astragalus hoffmeisteri* (Klotzsch) Ali (sect. *Pseudosesbanella*) and *Astragalus zadaensis* Podlech & L. R. Xu (sect. *Galegiformes*), both occurring in western Tibet. Detailed morphological differences among these three species are presented in Table 2.

**Table 2.** Main morphological differences among *Astragalus wuqiaensis*, *Astragalus hoffmeisteri*, and *Astragalus zadaensis*.

Characters	*A. wuqiaensis* sp. nov.	*A. hoffmeisteri*	*A. zadaensis*
Stem	Erect, 50–100 cm tall, forming dense clumps, densely pubescent at base, glabrescent toward apex.	Erect, up to 100 cm tall, flexuous, young shoots finely pubescent.	Erect, 30–40 cm tall, flexuous.
Stipule	ovate to triangular, 3–5 mm long, free, densely pubescent.	obliquely cordate-ovate, 3–13 mm long, connate at base, finely pubescent.	triangular, 6–7 mm long, free, sparsely white-ciliate.
leaflet	ovate to elliptic, 2–8 mm × 2–5 mm, apex emarginate with a short mucro, adaxial surface glabrous, abaxial surface and margin with white short-appressed hairs.	obovate, 4.5–11 mm × 2–8 mm, apex emarginate, both surfaces glabrous or sometimes sparsely pubescent along midvein.	obovate, 3–6 mm × 1.6–3 mm, apex emarginate, glabrous above, sparsely white appressed-hairy along the midvein beneath.
Calyx	tubular-campanulate, 6–7 mm long, densely pubescent.	tubular-campanulate, 4–5 mm long, glabrous to subglabrous.	campanulate, 2.5–3 mm long, subglabrous.
Corolla	yellow, 17–20 mm long.	pale purplish-red, 14 mm long.	yellow, 9–10 mm long.
Legume	1-locular, 30–40 mm × 10 mm, fusiform, slightly inflated.	1-locular, 11–12 mm × 4–8 mm, oblong, flattened.	1-locular, 20 mm × 4–5 mm, narrowly elliptic, slightly inflated.

##### Description.

**Figure 2.**
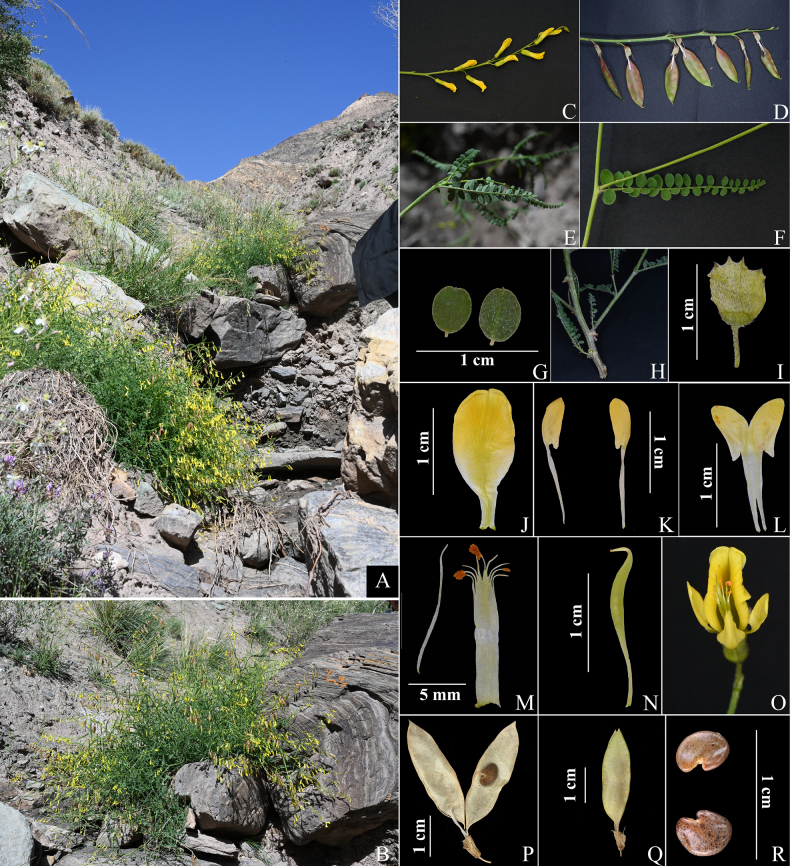
*Astragalus wuqiaensis*. **A**. Habitat; **B**. Whole plant; **C**. Inflorescence; **D**. Infructescence; **E**. Abaxial surface of compound leaf and stipule; **F**. Adaxial surface of compound leaf; **G**. Leaflets; **H**. Stem base; **I**. Calyx; **J**. Standard; **K**. Wings; **L**. Keel; **M**. Stamens; **N**. Pistil; **O**. Flower; **P**. Interior of the pod; **Q**. Pod; **R**. Seeds. Photographs by Zhao-Ping Yang and Si-wei Zeng.

**Figure 3.**
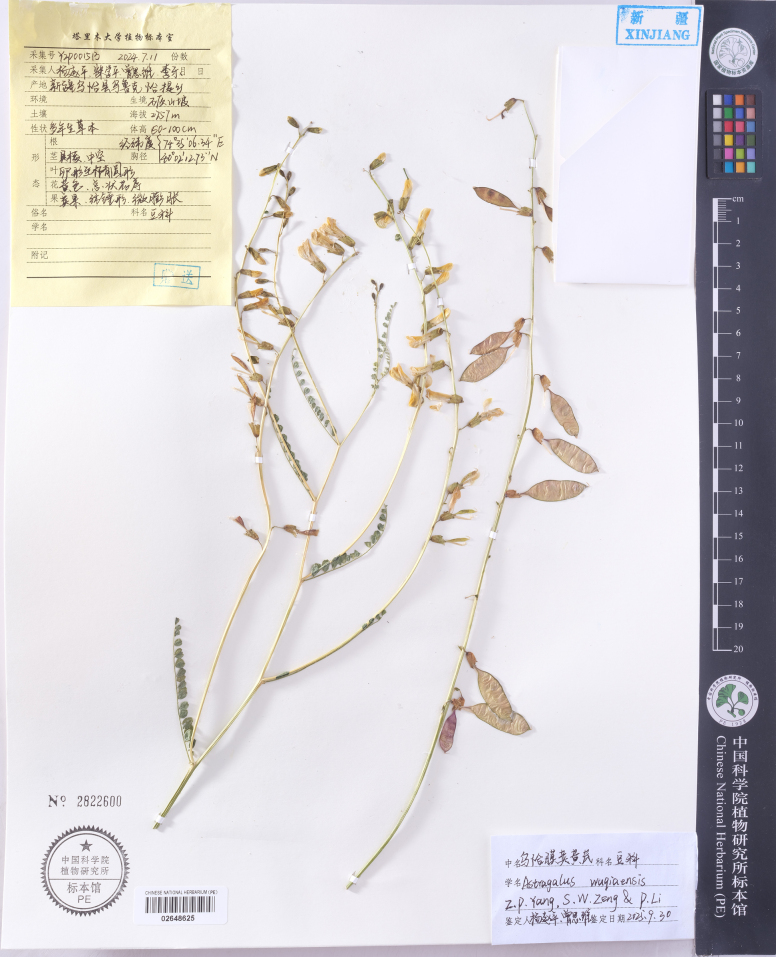
Holotype specimen of *Astragalus wuqiaensis* (PE02648625).

Perennial herb, 50–100 cm tall. ***Stems*** erect, much-branched, hollow, ridged, densely covered with white stiff hairs at base, sparsely covered with white stiff hairs on upper parts, with both white and black stiff hairs intermixed at junction with petiole. ***Stipules*** ovate, 3–5 mm long, glabrous on both surfaces, margin with white stiff hairs. ***Leaves*** odd-pinnate, 4–8 cm long; petiole ca. 1 cm long, with short white appressed hairs, intermixed with black appressed hairs at base; leaflets 9–20 pairs, 2–8 mm × 2–5 mm, ovate to elliptic, apex emarginate with a short mucro, base suborbicular to cuneate, adaxial surface glabrous, abaxial surface with white short-appressed hairs, margin with white short-appressed hairs; petiolule ≤ 1 mm, with white short-appressed hairs. ***Inflorescence*** an axillary raceme, lax, with numerous nodding flowers; peduncles conspicuously exceeding leaves, with white appressed hairs; bracts lanceolate, 2–3 mm, strigose on both surfaces; pedicels 3–7 mm, strigose. ***Calyx*** tubular-campanulate, 6–7 mm × 3–4 mm, external surface with mixed white and black strigose hairs, teeth short, triangular, less than 1 mm long, calyx tube oblique at orifice. ***Corolla*** yellow; standard 17–20 mm long, blade suborbicular, apex emarginate, base attenuate into a 4–5 mm claw; wings slightly shorter than standard, blade oblong, ca. 7 mm long, base with a short auricle, claw ca. 10 mm long; keel shorter than wings, blade with a short auricle, ca. 6 mm long, claw ca. 10 mm long; stamens diadelphous (9+1); ovary 1-locular, glabrous, slenderly long-stipitate, stigma without tufted hairs. ***Legumes*** 1-locular, membranous, fusiform, slightly inflated, *ca*. 3–4 cm × *ca*. 1 cm, stipe extending beyond calyx tube, up to 1 cm long. ***Seeds*** reniform, 4–5 mm × 3–4 mm, testa brown, smooth, with black spots (Figs 2, 3).

##### Type.

China • Xinjiang: Wuqia County, Ulugqat Township, 40°02'12.7280"N, 74°35'06.3416"E, elev. 2757 m. 11 July 2024, fl & fr. *Zhaoping YANG, Siwei ZENG et al. YZP001513* (Holotype: PE02648625!) (Fig. 3). China • Xinjiang: Wuqia County, Jigen Township, 40°14'37.39"N, 75°13'37.73"E, elev. 2857 m. 15 July 2021, fl & fr. *Zhaoping YANG, Shiqiang SONG*. YZP000257 (Paratype: TARU28021!).

##### Etymology.

The specific epithet *wuqiaensis* derives from the type locality of the new species.

##### Distribution and habitat.

**Figure 4.**
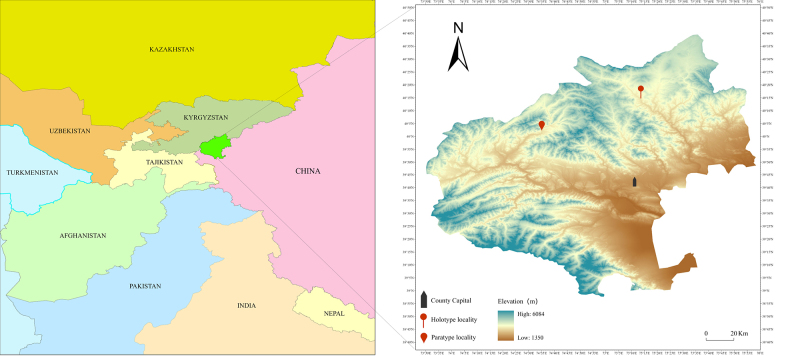
Distribution map of *Astragalus wuqiaensis*.

This species is currently known only from Ulugqat Township and Jigen Township, Wuqia County, China (Fig. 4). In Ulugqat Township, it occurs on arid, rocky slopes at ca. 2,700 m elevation along watercourses, associated with perennial herbs (e.g., *Orobanche amoena* C.A.Mey., *Artemisia* spp., etc.) and other xerophytic vegetation. In Jigen Township, it grows in dry meadows at ca. 2,800 m.

##### Phenology.

Flowering from June to July; fruiting from July to August.

##### Vernacular name.

We propose a Chinese name, Wū qià Mò jiá Huáng qí (Chinese pronunciation), 乌恰膜荚黄芪 (Chinese name).

##### Conservation status.

Currently, *A. wuqiaensis* is known only from two isolated populations ca. 70 km apart. Wuqia County is located in a remote, mountainous border region with limited accessibility, resulting in incomplete floristic survey coverage. Moreover, the county adjoins Kyrgyzstan, and the two areas share a substantial number of plant species, making a potential transboundary distribution of this species plausible. Owing to the currently insufficient distribution data, an accurate assessment under IUCN criteria is not feasible. Therefore, we recommend that it be provisionally classified as Data Deficient (DD).

## Discussion

Morphologically, sect. *Cenantrum* is diagnosed by free stipules, yellow to purplish-red flowers in lax racemes with elongated peduncles, stipitate ovaries, and unilocular, membranous legumes. The new species *A. wuqiaensis* possesses all these characteristics, supporting its placement in sect. *Cenantrum*. Furthermore, phylogenetic analysis of nrITS sequences strongly supports its placement within the core clade of sect. *Cenantrum* (BS = 96%, PP = 1.0). Consequently, both morphological and molecular evidence confirm that this taxon is a bona fide member of sect. *Cenantrum*. Within the section, *A. wuqiaensis* is distinguished by its ovate to elliptic leaflet shape, combined with its conspicuously small leaflets (2–8 mm × 2–5 mm). These are even smaller than those of *A. tokachiensis* (5–11 mm × 3–6 mm), which previously had the smallest leaflets in the section.

In our phylogenetic reconstruction, *A. muliensis* (currently classified in sect. *Cenantrum*) was unexpectedly resolved outside the sect. *Cenantrum* clade. Examination of “Flora Reipublicae Popularis Sinicae” (Ho and Fu 1993) and Plants of the World Online (POWO 2025) confirmed it as a Chinese endemic (distributed in Xizang, Sichuan, and Yunnan provinces). Notably, Ho and Fu (1993) state: “According to the protologue, this species appears to belong to sect. *Melilotopsis*; however, it is provisionally maintained in sect. *Cenantrum* due to the compilers’ inability to examine type specimens.” Our molecular phylogenetic analysis provides strong support (BS = 99%) for a monophyletic relationship between *A. muliensis* and *A. melilotoides* (a representative species of sect. *Melilotopsis*), thereby validating the original taxonomic inference that it belongs to sect. *Melilotopsis*.

## Supplementary Material

XML Treatment for Astragalus wuqiaensis

## Additional information

### Use of AI

AI tools were used only to polish the English language. No AI assistance was involved in data analysis, study design, or content generation.

### Author ORCIDs

Si-Wei Zeng https://orcid.org/0009-0002-5472-3004

Kai-Wen Jiang https://orcid.org/0000-0002-5917-1846

Cheng-Wen Luo https://orcid.org/0009-0003-2560-2147

Pan Li https://orcid.org/0000-0002-9407-7740

Zhao-Ping Yang https://orcid.org/0000-0001-5096-1051

### Data availability

All of the data that support the findings of this study are available in the main text.
